# Ongoing Processes in a Fitness Network Model under Restricted Resources

**DOI:** 10.1371/journal.pone.0127284

**Published:** 2015-05-18

**Authors:** Takayuki Niizato, Yukio-Pegio Gunji

**Affiliations:** 1 Faculty of Engineering, Information and Systems, Tsukuba University, Tsukuba, Ibaraki, Japan; 2 School of Fundamental Science and Engineering, Waseda University, Tokyo, Japan; UNIVERSITY OF LAUSANNE, SWITZERLAND

## Abstract

In real networks, the resources that make up the nodes and edges are finite. This constraint poses a serious problem for network modeling, namely, the compatibility between robustness and efficiency. However, these concepts are generally in conflict with each other. In this study, we propose a new fitness-driven network model for finite resources. In our model, each individual has its own fitness, which it tries to increase. The main assumption in fitness-driven networks is that incomplete estimation of fitness results in a dynamical growing network. By taking into account these internal dynamics, nodes and edges emerge as a result of exchanges between finite resources. We show that our network model exhibits exponential distributions in the in- and out-degree distributions and a power law distribution of edge weights. Furthermore, our network model resolves the trade-off relationship between robustness and efficiency. Our result suggests that growing and anti-growing networks are the result of resolving the trade-off problem itself.

## Introduction

Many evolving network models are based on the well-known property of preferential attachment [1, 2, 3]. We regard the main rule for preferential attachment in evolving network models as being constructed from at least the following two working concepts. (i) A new node is added to the network at every step, and (ii) a fixed number of edges are connected to existing nodes that have more connections than others (i.e. the probability of connecting a new edge between the new node and an existing node is proportional to the number of edges already connected to the existing node). Rule (ii) in particular suggests that new edges from new nodes preferentially attach to old nodes when these existing nodes have many links (the so-called rich-get-richer effect). For this reason, the set of rules (i) and (ii) is called preferential attachment. It has already been found that the heterogeneous connectivity of the network emerges from this preferential attachment. The scale-free network structure, in particular, is considered a ubiquitous property of the structure of many real networks. Preferential attachment is a good tool for determining the power laws in the structure of networks [2, 3].

The concept of preferential attachment has been criticized by many researchers [4, 5, 6, 7, 8, 9]. Mandelbrot, for example, criticized the mechanical aspect of preferential attachment (more specifically, the preferential attachment-like models proposed in the past) and pointed out the lack of any underlying mechanism [4]. We thus analyze the method behind the concept of preferential attachment. We point out that there are two hidden assumptions behind the two rules that we listed. One is that a newly added node can make a quick overall observation of information about the whole network because otherwise it would need to know the degree distribution of a given network in advance in order to distinguish the more popular nodes from the others. The other is the assumption that the number of nodes and edges in a given network always increases because the preferential attachment algorithm always adds a new node and a fixed number of edges to the network.

The first hidden assumption has been pointed out by many researchers [5, 6, 7, 8, 9]. Researchers who have tried to resolve this problem have introduced internal dynamics to nodes and added edges by many different processes. Although there are many approaches to this problem, we consider that a common approach is to limit the selection of nodes when using preferential attachment. In other words, the nodes selected from the population are restricted by taking into account the activation of the node, aging, and locality selection, which is called a “local world” [5, 6, 7]. This kind of restriction on the selection of nodes prevents us from presuming global information about the degree distribution of nodes. All a new node needs to do is to seek more popular nodes from this limited set of nodes. Of course, these approaches are an extension of preferential attachment.

Unlike the first hidden assumption, the second one is seldom discussed. Real networks such as the World Wide Web (WWW) [10, 11] and social networks [12, 13, 14], are indeed expected to grow virtually without limit. However, if we look at other real networks, we discover that cases of networks that grow forever are rare. For example, ecological networks have at most two hundred nodes (species) and a thousand edges (such as the flow of carbon or energy) [15, 16, 17]. This fact also applies to airport transportation networks [18] and protein reaction networks [19]. In these cases, we cannot expect an ever-growing network like the WWW. With respect to the WWW, the important point of the listed network is the temporal dynamics of the networks, that is, the increases and decreases in the number of nodes and edges, since the nodes and edges in ecological networks and other networks change with time due to the effect of external perturbations such as extinction events.

However, we need to point out here the radical assumption of the evolving network model. The radical assumption in preferential attachment is that most models admit an asynchronous update rule in their algorithms [1, 2]. In other words, there is no change in the network dynamics without the addition of new nodes and selected existing nodes. By using an asynchronous updating rule, we are able to obtain perfect information about network structures, such as the degree distribution, and to construct algorithms for preferential attachment. We feel that this problem is critical because protein reaction networks and networks of energy flows between species involve events on many timescales, with these multi-scale processes occurring at the same time. Furthermore, the study of network theory is motivated by attempting to understand these many possible processes as a whole system.

This methodological problem is also relevant for relatively small networks. It is well-known that flows of network have multiple time scales in relatively small networks (e.g. ecosystems) [20]. In addition, it is known that the existence of a weak link in a network contributes to network stability [21]. All these facts suggest that the assumption of asynchronous updating is not appropriate, particularly for finite and small networks, because we cannot ignore these observed facts in relatively small networks. If we take this problem seriously, we cannot expect preferential attachment to function in a network model. Preferential attachment therefore needs to be modified when constructing an evolving network model.

We thus propose a new model for evolving networks. Our network model is constructed by individuals who have their own fitness which is determined by their environment. Each individual belongs to one state (this corresponds to being a node in the network). Each individual tries to raise its own fitness and change its state. These state transitions of individuals create a weighted state transition network which dynamically changes with time. Our network model exhibits an asymmetrical relationship between the in-degree and out-degree distributions. Furthermore, the dynamical aspect of our evolving network model leads us to consider a developmental process in the network (growing and decaying) with time.

The paper consists of the following sections. In section 2, we explain the algorithm used in the model and how the state transition network is constructed. Our model uses lattice theory, which is often employed in computer science. The basic notion of lattice theory is detailed in S1 Text. In Section 3, we analyze the evolving network in our model such as in terms of clustering coefficient, degree distribution, and the trade-off relation between the mean degree (MD; diversity of states) and variance of edge weights (the overall gain in fitness). Finally, we discuss a developmental cycle in the network of our model.

## Methods

### Construction of the Model

Before we construct the network, we describe the underlying dynamics of the transition network. We assign each individual an *n*-bit binary string, and represent each individual by *s*_1_ *s*_2_ *s*_3_…*s*_*n*_ and *s*_*i*_∈{0, 1} for all *i*∈{1, 2, …, *n*}. For brevity, we denote *s*_1_ *s*_2_ *s*_3_…*s*_*n*_ by 〈*s*〉_*n*_. Each bit string represents a state to which each individual belongs. If two individuals have the same bit string, then these two individuals belong to the same state. In our model, each individual tries to adapt to an environment, which is represented by the target bit strings (we denote 〈*t*〉_*n*_, i.e. *t*_1_ *t*_2_ *t*_3_…*t*_*n*_), through its interactions. We assign the values of the target bit strings at random. The adaptive process of each state in our model is the process by which each individual attempts to change its state into the target bit string 〈*t*〉_*n*_. Because of the existence of the target bit strings, we can define the fitness of each individual. The fitness measures the degree of adaptation of an individual to its environment. In other words, if the bit string of an individual matches the target bit string well, then it has high fitness for the environment. The fitness of each individual is therefore defined by the Hamming distance from the target. Mathematically, we compute the fitness of each individual as follows.

#### Definition 1

(*Fitness Bit String*) Let 〈*s*〉_*n*_ and 〈*t*〉_*n*_ be *n*-bit strings where 〈*s*〉_*n*_ is the state to which an individual belongs and 〈*t*〉_*n*_ is the target. Let *k* be a hidden digit in the target bit string.

For 1≤*j*≤*n*,

$$b_{j}=\{\begin{array}{c} \;0\;(\text{if}\;s_{j}=t_{j}\;\text{and}\;j\neq k)\\ \;1\;(\text{if}\;s_{j}\neq t_{j}\;\text{and}\;j\neq k)\\ \;0\;\text{or}\;1\;\text{randomly}\;(\text{otherwise}) \end{array}$$

We call 〈*b*〉_*n*_ the fitness bit strings for the target.

We therefore have three distinct types of bit strings. The first type is bit strings representing the state of an individual (denoted 〈*s*〉_*n*_). The second type is bit strings representing the fitnesses of individuals (denoted 〈*b*〉_*n*_). The last type is bit strings representing a target (denoted 〈*t*〉_*n*_). The main difference among them is that the first two (state 〈*s*〉_*n*_ and fitness 〈*b*〉_*n*_) types of bit string change over time, whereas the target bit string 〈*t*〉_*n*_ is fixed throughout each trial.

In particular, fitness bit strings can be compared with each other quantitatively. When the number of 1s in the sequence 〈*b*〉_*n*_ is higher, the fitness represented by the string is higher (for instance, 100 ≤ 111). The order relation between two binary bit strings is defined by 〈*a*〉_*n*_ ≤ 〈*b*〉_*n*_ if *a*_*i*_ ≤ *b*_*i*_ for all *i* ∈{1, 2, …., *n*}. An important of property of partial ordered sets is the possible existence of pairs of elements that are incomparable (for instance, 101 and 010 are incomparable because 101 is larger than 010 at the first digit but smaller than 010 at the second digit).

The concept of hidden digits in Definition 1 represents imperfect knowledge about the environment. In other words, each individual never has perfect knowledge about its environment. Each individual can only observe *n*-1 bits of an *n*-bit target string. The fitness value of the hidden position is determined randomly (zero or one). The position of the hidden digit changes randomly from the first digit to *n*th digit at every step. If the position of a hidden digit is *k* (i.e. the *k-*th digit in a target bit string 〈*t*〉_*n*_) at time *t*, each individual can observe the target bit string *t*_1_ *t*_2_…*t*_*k*-1_ *t*_*k*+1_…*t*_*n*_. Each individual tries to adapt to the uncertain environment by changing its state. Since an individual tries to raise its own fitness, it leaves its states the same (*s*_*j*_^*t+*1^ = *s*_*j*_^*t*^) if it has high fitness digits (*b*_*j*_ = 1) and changes its states randomly (*s*_*j*_^*t+*1^ = 0 or *s*_*j*_^*t+*1^ = 1) if it has low fitness (*b*_*j*_ = 0). The idea of using a fitness measure for each state in a network is not new [22, 23, 24, 25].

Unlike previous fitness models, we assume no particular distribution function for fitness [22, 23]. In these models, the fitness information for each node is completely given. In contrast, the positioning of fitness in our model is utterly different because each individual lacks complete information (i.e. hidden digit) about its own fitness. Each individual tries to estimate its own fitness from incomplete information [26]. Taking this into account, the main difference in the attitude toward fitness is whether the fitness is given passively (fitness is given to a node as perfect information) or actively (fitness is given to a node as imperfect information). We construct this fitness during the development of the network as described in a later section.

Next we discuss the fitness dynamics in terms of the interactions between states. We represent the fitness dynamics by using a quotient lattice from lattice theory (details of the definition of the quotient lattice are described in S1 Text). By using a quotient lattice, the fitness of each individual is never only determined by a local estimate, but also by a global estimate. The concept of quotient lattice is not difficult. The quotient lattice is a way of grouping a complete lattice (S1 Text). In lattice theory, a lattice is defined as a partially ordered set which is closed under the operations of join and meet. Roughly speaking, this implies the existence of bottom and top elements in a partially ordered set. In terms of fitness, the top element means high fitness overall. If an individual attains the highest fitness, there is thus no need to change state. Although it is generally difficult for individuals to achieve the highest fitness, we found that our algorithm made it possible for individuals to easily obtain the highest fitness despite the existence of individuals whose fitness is *not* the highest.

According to Theorem 1.11 (see S1 Text), we can obtain a unique quotient lattice if we take one element from a lattice. This grouping result, that is, the quotient lattice, corresponds to the global fitness. Generally, fitness cannot be determined only by local estimation (comparing with only a target bit string), but requires taking the global context into consideration. In this respect, a lattice for fitness corresponds to a global structure of fitness, and a quotient lattice for fitness corresponds to a global estimation. Mathematically, we can represent the fitness of each individual in a global context as follows.

#### Definition 2

(*Fitness Being Induced by a Quotient Lattice*) Let *L* be a lattice of *n*-bit strings, *J* be an ideal and *θ* (*J*) be a congruence on lattice *L*. For 1≤*i*≤*N*,

$${\left\langle b\right\rangle }_{n}^{i,t}\leftarrow \vee \{{\left\langle x\right\rangle }_{n}^{k,t}\in L^{t}|{\left\langle x\right\rangle }_{n}^{k,t}\in {\left[{\left\langle c\right\rangle }_{n}^{j,t}\right]}_{\theta (J)}\;\text{such that}\;{\left\langle b\right\rangle }_{n}^{i,t}\in {\left[{\left\langle c\right\rangle }_{n}^{j,t}\right]}_{\theta (J)}\}$$

We denote the substitution operation by ← and join by v. *N* is the total number of individuals. [⟨*b*⟩_*n*_]_*θ*(*J*)_ means an element in a quotient lattice being derived from an ideal *J* and its congruence *θ* (*J*). Therefore, a binary bit string of fitness blocks is replaced by the *largest element* in the congruence to which it belongs.

Details of the definition of an ideal, congruence, and join are listed in S1 Text. We only point out here that each individual takes the largest element to which it belongs in a given quotient lattice. Taking the largest element (i.e. fitness) against the original fitness estimated by local incomplete information means that each individual overestimates its own fitness when we take into account the global context for fitness estimation. However, this overestimation never indicates that the fitness actually increased. The overestimation of fitness is a kind of *error* induced by considering the global context. Each individual uses this overestimated fitness to change its state. The main effect of fitness overestimation is to suppress the degree of change in an individual state. For example, assuming we have a 3-bit string 100 (the state of an individual) and its fitness 001 (the fitness of an individual), this individual randomly flips the corresponding digits, that is, the 10 of 100. There are thus four possible changes (from 100 to 100, 110, 010, or 000). If overestimation of the fitness of this individual occurs due to the global context, such as if the fitness becomes 011, then there are only two patterns of change (from 110 to 010 or 110).

We briefly summarize the algorithm from our model.

1. Generate *N* = 2^*n*^ *n*-bit strings 〈*s*〉_*n*_^1,0^,〈*s*〉_*n*_^2,0^, …〈*s*〉_*n*_^*N*,0^ and a target *n*-bit string 〈*t*〉_*n*_ randomly. The target is fixed through one trial. Note that these bit strings may be bitwise-identical (i.e. 〈*s*〉_*n*_^*i*,0^ = 〈*s*〉_*n*_^*j*,0^ but be distinct as individuals *i* and *j*).
2. Randomly select a hidden digit of the target from the set {1, 2, …, *n*}. A fitness is formed from a set of *n*-bit strings *B* ^*t*^ = {〈*b*〉_*n*_^1,*t*^, 〈*b*〉_*n*_^2,*t*^, …, 〈*b*〉_*n*_^*N*,*t*^} using Definition 1.1.
3. Create a lattice *L* ^*t*^ from this *B* ^*t*^.
4. Select one element 〈*b′*〉_*n*_^*t*^ in the lattice *L* ^*t*^ and create an ideal ↓〈*b′*〉_*n*_^*t*^.
5. Construct a quotient lattice from the ideal *J* = ↓〈*x*〉_*n*_^*t*^.
6. Substitute new binary bit strings of fitness blocks using Definition 2.
7. Change each *n*-bit string using fitness blocks. For 1≤*j*≤*n*,

$$s_{j}^{i,t+1}=\{\begin{array}{c} s_{j}^{i,t}\;(\text{if}\;b_{j}^{i,t}=1)\\ 0\;\text{or}\;1\;\text{randomly}\;(\text{otherwise}) \end{array}$$

Construct a new set of binary bit strings for species *B* ^*t*+1^ = {〈*s*〉_*n*_^1, *t*+1^, 〈*s*〉_*n*_^2, *t*+1^, …, 〈*s*〉_*n*_^*N*, *t*+1^}.

The sequence of steps (2)-(7) forms one time step of a trial. Repeating steps (2)-(7), we can observe the evolution of the state and the lattice. Details of the algorithm are listed in S1 Text. The state in our model thus forms a lattice from local incomplete information, with fitness re-estimated from the global estimate, which is derived from a quotient lattice. The important point is that an individual never asynchronously updates its state. All individuals estimate their fitness and change state at the same time.

### Creating a Transition Network

**Fig 1:**
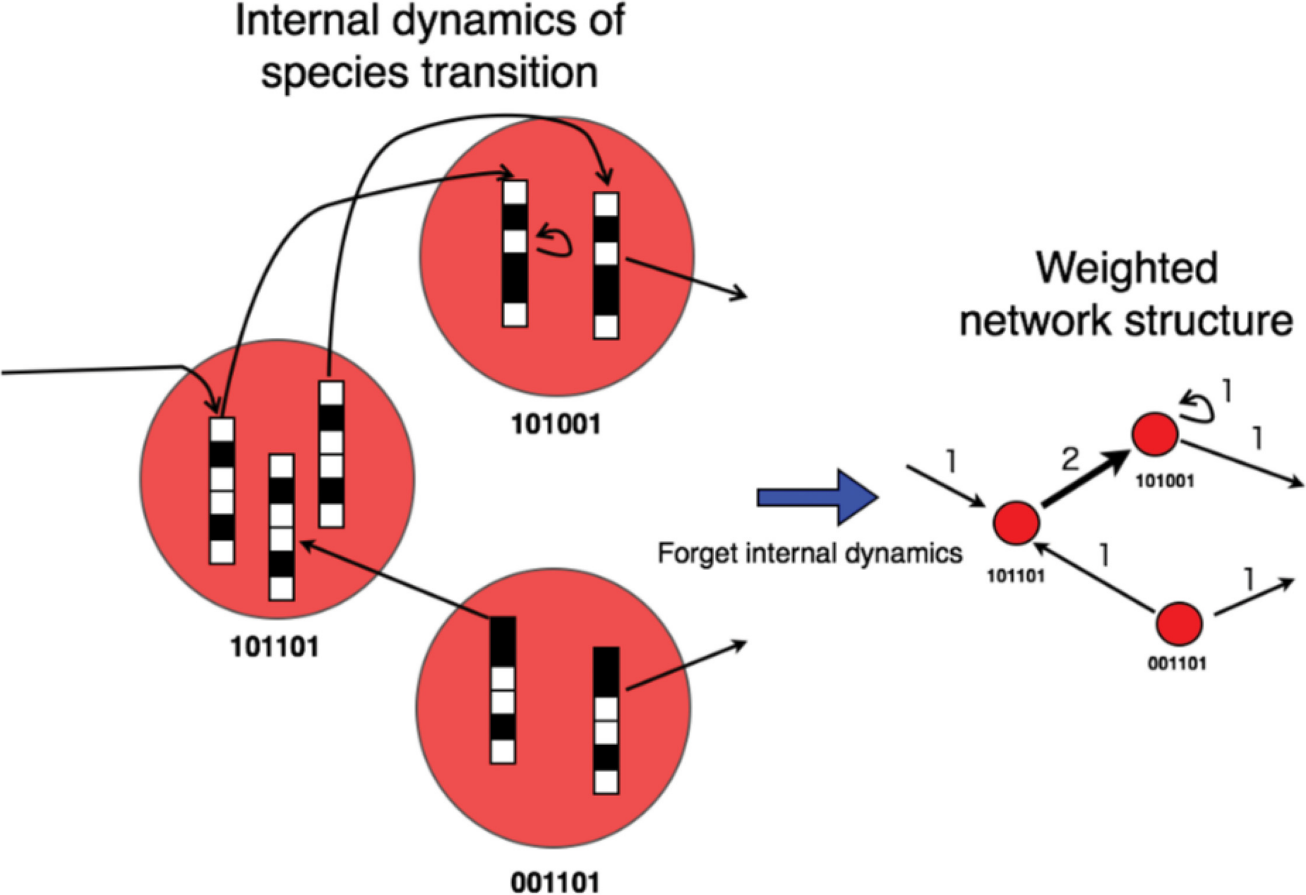
Example of the state transitions of all individuals. Constructing a network from the state transitions of all individuals. In this example, the network is constructed in one step from the state transition shown on the left. Bit strings (colored black for 0 and white for 1) represent individuals and red circles represent states (i.e. nodes). Individuals who have the same state are shown in the same circle. Arrows indicate transitions of individual induced fitness. The figure on the right represents the state transition network. Numbers above edges indicate the weights of the edges. The network contains a self-loop. Note the number of individuals (i.e. 2^*n*^) never change with time but only the number of states.

In the previous section, we explained how each individual attempts to raise its fitness and change its state, which corresponds to the nodes in this section. By using this algorithm, we can construct a weighted transition network. In this network, nodes represent states (the states of the individuals) and edges represent transitions between states. The weight of each edge is given by the number of transitions in a certain interval. Fig 1 shows an example of the construction of a state transition in a network. The edge is formed by actual transitions in a certain interval (one step in Fig 1). The number above each edge represents its weight. From this example, it can be found that the sum of the weights of all edges is always fixed because all individuals change state every step. Therefore, if there are 32 individuals, then there are 32 state transitions which correspond to the edges. This can be expressed mathematically as follows:

$${\left\|<s{>}_{n}\to <s\prime {>}_{n}\right\|}_{w}=\sum_{t=t_{1}}^{t_{2}}\left|\left\{<s^{i}{>}_{n}^{t}\to <s^{i}{>}_{n}^{t+1}\;\text{occur at time}\;t\;\text{when}\;<s{>}_{n}=<s^{i}{>}_{n}^{t}\;\text{and}\;<s\prime {>}_{n}=<s^{i}{>}_{n}^{t+1}\right\}\right|$$

where ||-||_*w*_ means the weight of the edge which corresponds to the state transition 〈*s*〉_*n*_^*t*^→〈*s*〉_*n*_^*t*+1^ and *t*_2_-*t*_1_ is a fixed interval of time. If we take enough time intervals (200 steps in this paper), we can obtain a fully connected directed weighted network (in this case, the total weight of edges is 200*N*. *N* is the total number of individuals).

We note that the transition network never satisfies the transitive property (if A implies B and B implies C, then A implies C) of edges. The possibility of transition exists in the network where a triangle relationship exists. Because of the fixed sum of weights, the weighted network suggests a trade-off relationship between the gain of all individuals (which corresponds to high overall fitness) and the resource distribution (which corresponds to a high diversity of species). This problem is discussed in a later section.

## Results

### Cluster Coefficient and Local Cluster Coefficient

Before we analyze the properties of the network of our model, we construct a control model with behavior that can be controlled by tuning one parameter. Since the details of the algorithm of the control model are listed in S1 Text, at this point we note that the concept of the model is to have a way of measuring fitness which can be controlled by one parameter. This parameter controls the error rate. The error rate is defined in terms of the probability *μ* that a digit that is the same in an individual as in the target bit string changes value. For example, we again consider three-bit strings (with 100 for the state and 011 for the fitness). Each of the "1" digits in this fitness (i.e. the last of two 1s) changes to 0 with probability 1- *μ*. The error rate therefore increases as *μ* increases. Therefore, by tuning this parameter *μ*, we can control the behavior of the species to mimic our evolving lattice (EL) model. Furthermore, because of this algorithm, high fitness states tend to become linked with each other without using any global information (fitness is determined by using only a target bit string). This model corresponds to a PA-like model under a finite resource environment. Compared with this control, the effect of using a lattice structure in our model becomes clearer.

**Fig 2:**
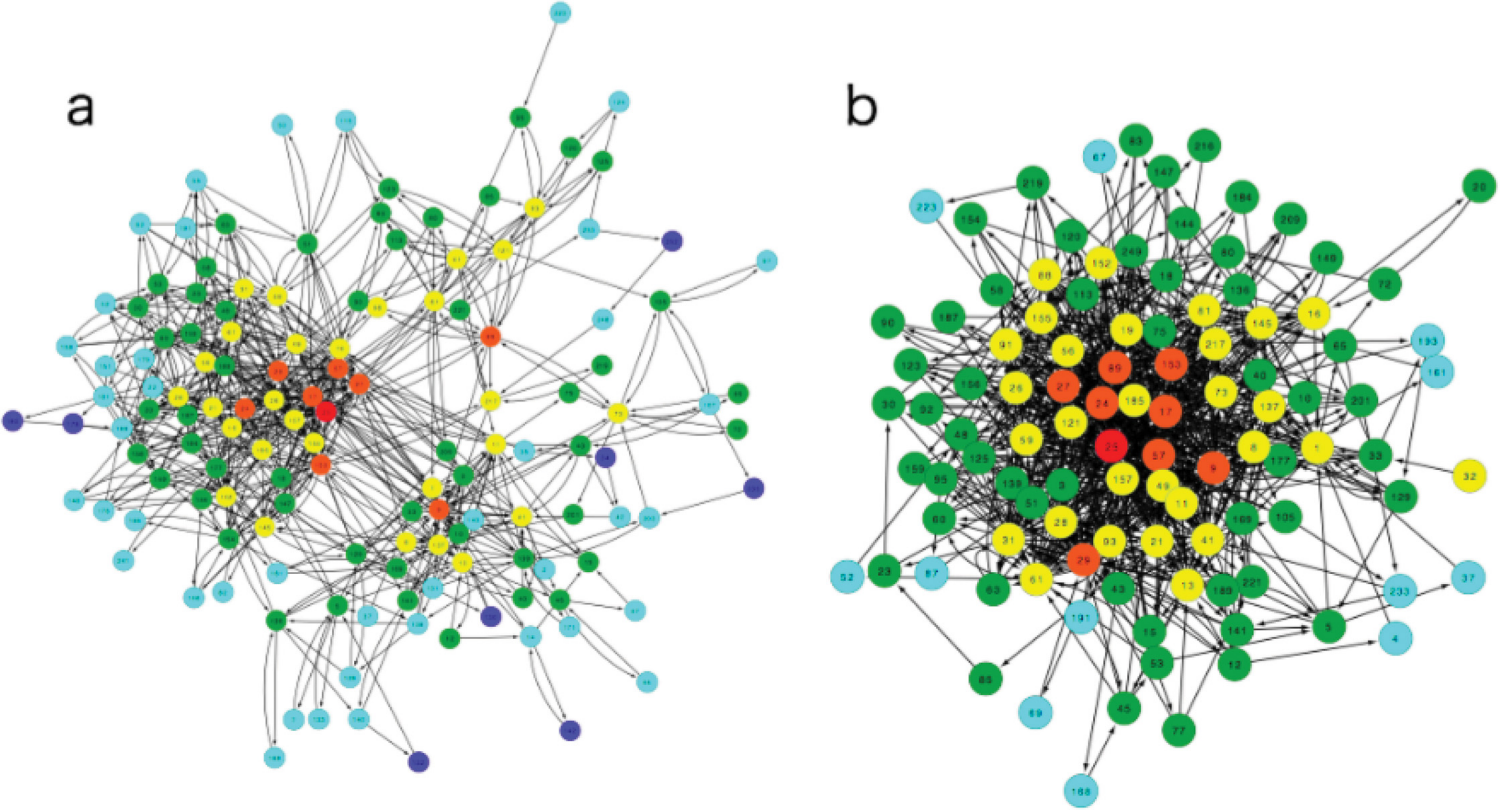
Examples of a network structure for the EL model (a) and the control (b). **(a)** The network of the EL network. Node colors represent the Hamming distance from the target bit string. Number in circles indicate the state. There are six colors, which are red (0), orange (1), yellow (2), green (3), light blue (4), and blue (5). The color of the target is red because the Hamming distance is zero. The global cluster coefficient and mean degree of the control network are much larger than those of our model. The number of nodes is always smaller than max possible nodes (i.e. 2^*n*^) because many low fitness states may not be selected. In this figure, the total number of node is 142 (EL model) and 168 (Control). **(b)** The control network (*μ* = 0.005). Colors are the same as Fig. 2A.

Fig 2A and 2B show two networks from our EL model and one example of the control model (parameters are fixed at *μ* = 0.005). All data in this paper are averages from over 100 times trials for the EL model and the control. As is shown in a later section, the state of the control model when the parameter μ is 0.005 is near the EL model compared with other parameter values (see S1 and S2 Figs). We point out here the difference can be observed as a network structure (i.e. cluster coefficient). First, the global cluster coefficient of the control is four times larger than that of our model (*C* = 0.133 ± 0.020 for the EL model and *C* = 0.474 ± 0.028 for the control). In the same way, the mean degree of the control is two times larger than that of our model (MD = 5.00 ± 0.50 for the EL model and MD = 9.25 ± 0.32 for the control). This fact suggests the possibility that the state transition is restricted in the EL model. Compared with the EL model, the state transitions of the control are only controlled by one parameter. Therefore, the transition of species is determined statistically and never depends on the global context of the whole process. However, state transitions in the EL network are determined not only in a statistical way, but also from the lattice structure. Owing to the lattice structure (or replacing each fitness with the top element of the quotient lattice), the possibility of state transitions never becomes free like in the control model.

**Fig 3:**
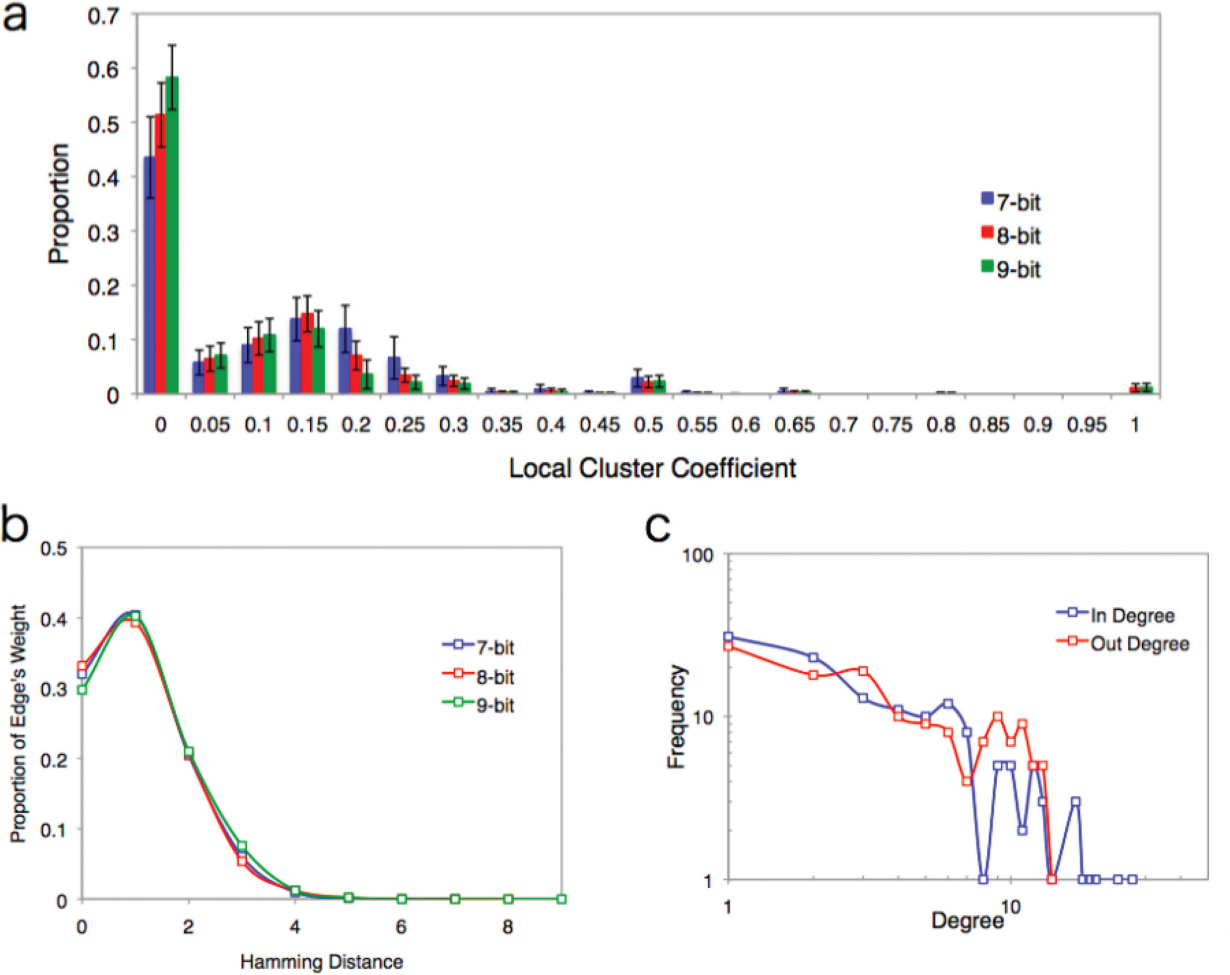
Details of data for the EL network. **(a)** Distribution of the local cluster coefficient in the EL network. The horizontal axis corresponds to the local cluster coefficient (binned at 0.005) and the vertical axis corresponds to the proportion. **(b)** The proportion of edge weights for each Hamming distance. A large proportion of edge weights are concentrated within 3 bits. This tendency is the same in all cases. **(c)** The in-degree and out-degree degree distributions for the EL network. Both graphs show an exponential decay and the same scaling parameter λ_in_ = 0.226 ± 0.026 and λ_out_ = 0.226 ± 0.026. We use a maximum likelihood estimation method with a discrete distribution [27, 28].

While we have already mentioned the global cluster coefficient *C*, we also examine the detail of the clustering structure around each node because the global cluster coefficient never refers to the local clustering structure of the network. Fig 3A shows the distribution of the local cluster coefficient of the EL network (binned 0.005). Although the EL network has a relatively small cluster coefficient, it has a high local cluster coefficient of around 1.0. This high local cluster coefficient is distributed around the target state. Around the target state, which has a highest fitness, the EL network never only has a high local cluster coefficient, but also has high weighted edges. In Fig 3B, the weight distribution of edges in the state as classified by Hamming distance from the target are concentrated in small Hamming distance nodes (about 70% of a total weight of edge within 3 bits).

### Weight and Degree Distribution of the Evolving Lattice Model

Next we examined two frequency distributions for in-degree and out-degree because it is given that the network is directed. However, the shapes of the degree distributions are constructive (Fig 3C). It seems that the slope of in-degree (λ_in_) is steeper than that of out-degree (λ_out_), but the statistical values are the same, with λ_in_ = 0.226 ± 0.026 and λ_out_ = 0.226 ± 0.026 (the detail of statistical test are listed on Table 1). However, the distribution of the tail position (i.e. the highest degree in the EL network in a certain interval) of in-degree (max *k*_in_) is much larger than that of out-degree (max *k*_out_) (See Table 1). In other words, there is an asymmetrical relationship between the in-degree and out-degree distributions. This difference comes from the fitness algorithm of the EL network. Since each individual changes its state to increase its fitness, there are some nodes (state) that attract more edges than others. This popularity of nodes creates a ranking among all the nodes in the network. In contrast, the case of out-degree distribution is not related to the fitness algorithm of our model like the case of in-degree distribution. The out-flow and in-flow in the network obey the different underlying mechanism when the degree distribution is constructed. We note that low cluster coefficient and exponential decay of degree distributions are observed in the WWW [10, 11].

**Table 1:** Statistical data for an in-degree and an out-degree distribution.

	*λ*_*in*_ (mean±SD)	*λ*_*out*_ (mean±SD)	*t*-value	*p*-value
7-bits	0.234±0.031	0.234±0.029	-0.1613	0.872
8-bits	0.226±0.026	0.226±0.026	0.1223	0.902
9-bits	0.223±0.025	0.223±0.025	-0.0486	0.961
	max *k*_*in*_ (mean±SD)	max *k*_*out*_ (mean±SD)	*t*-value	*t*-value
7-bits	25.0±3.75	13.5±1.75	22035	<10^−16^
8-bits	24.9±3.76	13.5±1.76	22028	<10^−16^
9-bits	31.7±4.81	15.1±1.84	22201	<10^−16^

The above table means t-test for relation between an in-degree distribution $e^{-\lambda_{in}x}$ and an out- degree distribution $e^{-\lambda_{out}x}$. The bellow one means Mann-Whitney U test for the highest degree for those degree distributions, that is, $\max\;k_{*}=\max\{k|e^{-\lambda_{*}x}>0\}$.

**Fig 4:**
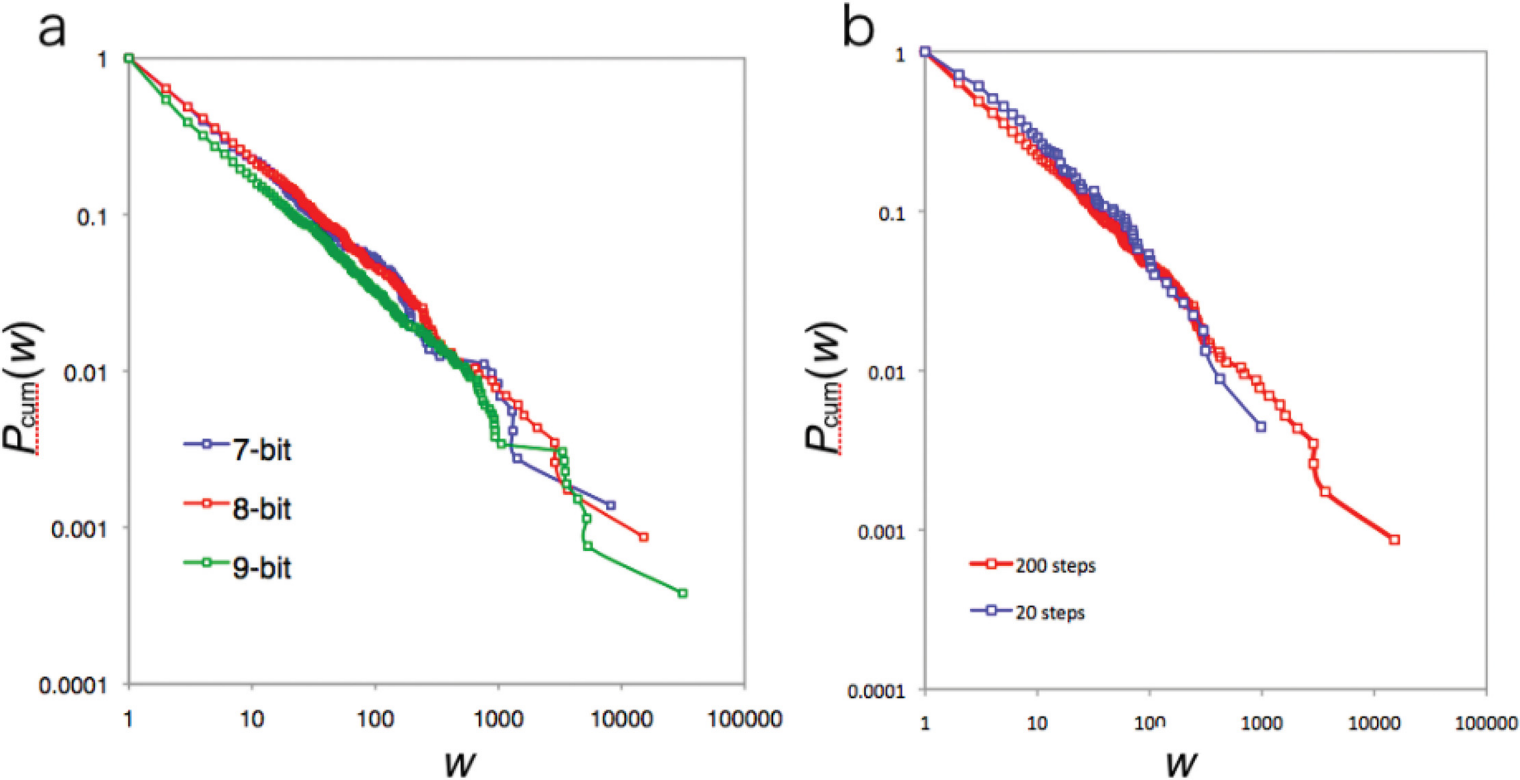
Cumulative Distribution of edge weights. **(a)** Cumulative distribution of edge weights. Colors correspond to the number of bits. All graphs exhibit a power law. The scaling parameter of the frequency distribution of this graph is about 1.5. **(b)** Cumulative distribution of edge weights for 20 steps interval and 200 steps interval. Both graphs follow a power law. The scaling parameter of the frequency distribution of this graph is about 1.5.

Next we investigate the frequency distribution of weights. Most real networks are weighted networks [18, 21, 27]. The examples of ecosystems and social communities, where weak links play an important role in the stability of the network, are often cited [21]. However, despite the importance of weighted networks, most studies focus on unweighted networks. Many researchers construct weighted networks by using a preferential attachment method, the same as unweighted networks [29, 30, 31, 32, 33]. As we discussed before, our model is not preferential attachment because the update rule is synchronous. Despite the synchronous update rule, it is not a given that time progress will occur at each individual because some individuals may change at time *t* and some individuals may remain in the same state (digits) at time *t*. In this network, the weight of an edge is measured by counting state transition events in a certain time interval (200 steps in this study). We examined the frequency distribution of edge weights in Fig 4A. The graph exhibits a power law distribution with a scaling exponent of 1.5. This power law behavior never depends on the number of bits (see Table 2). Additionally, the power law distribution of our model never depends on the length of the time interval (Fig 4B). If we take a 20 step interval, the species transition network never becomes an all connected network but exhibits the power law distribution for edge weights (see Table 2). This fact suggests that each state is already balanced in terms of weight to create the power law behavior as a whole before the all connected network is constructed. In this sense, we can also assume that the scale-free weighted network emerges as a result of each state trying to balance the amount of flow. This unconnected network in the middle of a process is natural because when we observe networks in nature, these networks are the result of long-duration processes. In ecosystems, for example, it is known that there are many time scales in each observed network (called slow channel and fast channel [20]).

**Table 2:** Data for a scaling parameter *α* of weight of power law with AIC test.

	Scaling Parameter *α*	AIC weights: *w*(*p*)
7-bits (200 steps interval)	1.55 (*N* = 25600)	1.00
8-bits (200 steps interval)	1.55 (*N* = 51200)	1.00
9-bits (200 steps interval)	1.65 (*N* = 102400)	1.00
8-bits (20 steps interval)	1.48 (*N* = 51200)	1.00

Scaling parameter α and Akaike information criterion (AIC) weights of power law for *w* ^−*α*^. *w* mean an edge’s weight.

### Trade-Off Relationship between Resource Distribution and Gain

In this section, we discuss the trade-off relationship between the resource distribution and gain in fitness. We consider that two properties may be important when we consider the efficiency and robustness of the weighted network. In network theory, the robustness of a network is measured by some researchers as ultraresilience against attacks [20, 34]. They define ultraresilience as the way in which the removal of nodes affects the global structure. However, ultraresilience is valid in non-weighted networks. In weighted networks, we take into account both the weight distribution and the edge distribution. The approach to this problem thus needs to be modified for weighted networks.

In weighted networks, in order to measure the robustness of a network, we select a mean degree and variance of edge weights in the network. The role of both quantities is clear. The role of mean degree corresponds to network robustness which we observe in non-weighted networks, while the role of variance of edge weights corresponds to the efficiency of the network.

The concept of robustness originally meant the fault tolerance to the removal of any one node from a given unweighted-network. In our model (a weighted network), taking away one node from a given network results in an inhibition of any transitions to this removed node. Because there are no corresponding nodes for these transitions, individuals whose destination state is this node will die (i.e. transition to an empty node). The removal of nodes from a network thus corresponds to the “extinction” of individuals who attempt to change their state to that node. Therefore, the meaning of robustness, which we discuss in this study, is defined as “the degree of removal rate that results in the removal of one node (i.e. state) from a given network”. When we restrict to the case of finite resources, this robustness corresponds to the “average degree” of a network. If the state transitions in a network are not concentrated in certain nodes, then other state transitions, which never go to these nodes, must occur in other states. This tends to increase the degree of the network overall. Thus, a high average degree network has a high robustness, whereas a low average degree network has a low robustness.

Next, the concept of efficiency in this study is defined as follows. “Efficiency” usually means the largest gain for the least effort. The gain in a weighted network means the fitness in this study. Therefore, the meaning of fitness in this paper is defined as “the degree of the total fitness in a given network”. From this definition, each individual needs to change its state to higher fitness state in order to increase the fitness and increase the efficiency of the network. This tendency results in a concentration on certain states that have high fitness. The weight distribution of heterogeneous edges thus increases (this tendency also means the average degree of the network becomes low). We measured the degree of heterogeneity as the variance of edge weights. We note here that the mean degree (i.e. robustness) and variance of edge weights (i.e. efficiency) in a network are in conceptual conflict with each other. If the mean degree of a network increases, the weights (in other words, the resources) of edges in the network distribute uniformly to each edge. An increase in the number of edges means a decrease in the weights of edges. In this case, the network gains robustness against external perturbations because the elimination of a node does not collapse the whole structure. By contrast, a decrease in the mean degree increases the heterogeneous weight distribution in the network. This results in increasing the overall fitness of the whole network.

**Fig 5:**
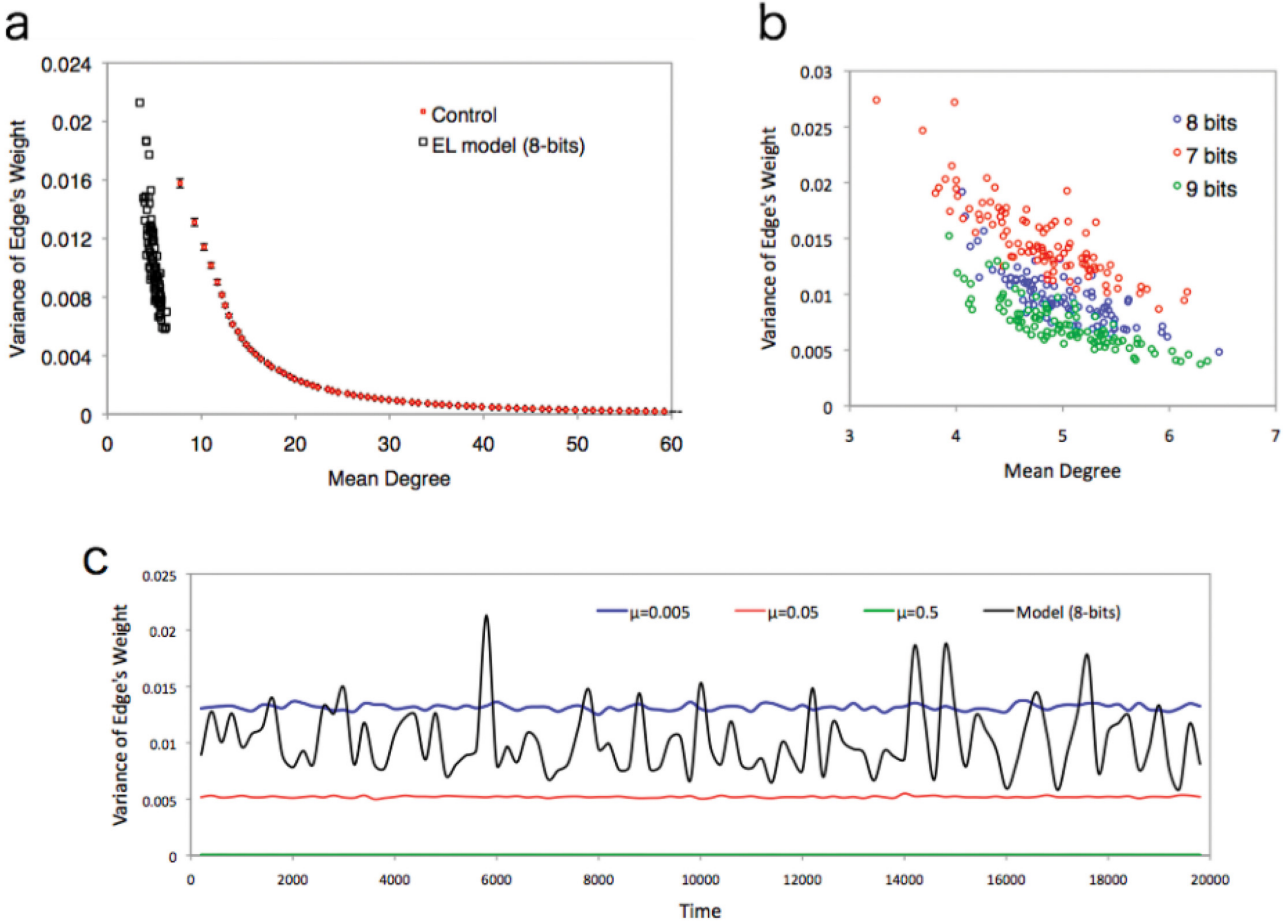
Trade of relationship between the mean degree and variance of edge weights. **(a)** Trade-off relationship between the mean degree and variance of edge weights. Weights are renormalized at each dot. Red indicates the control model and black indicates the EL model. Bars around the red dots show the standard deviation. **(b)** Negative correlation between the mean degree and variance of edge weights for each case (Peason correlation test, 7-bits: N = 87, *r* = –0.832, *p*<10^*–16*^; 8-bits: N = 87, *r* = –0.800, *p*<10^*–16*^; 9-bits: N = 97, *r* = –0.823, *p*<10^*–16*^). **(c)** The time series of variance of edge weights for the control (*μ* = 0.005, *μ* = 0.05 and *μ* = 0.5) and EL model (8-bits). Each value oscillates with time. However, compared with EL model, the fluctuation of the variance of the edge weights is very small.

The trade-off relationship between these two factors (i.e. robustness and efficiency) was confirmed by using the control model for parameter tuning. Increasing the value of *μ* suggests that the error increases for the fitness estimates of each individual. This tendency leads to the creation of a highly connected network because the error of fitness estimation increases the transitional possibility to many states. However, decreasing the value of *μ* reduces the error estimation. Therefore, a low value of *μ* suppresses false transitions due to errors. Instead, the weights of edges connected to correct states (corresponding to transitions to nodes near the target) increase. This gives the network a high heterogeneous weight distribution. Fig 5A shows the trade-off relationship between the degree of heterogeneity of the weights of the edges and the mean degree of the network (the parameter values *μ* are taken from 0.005 to 0.1 for every 0.005 interval). This graph shows that the distribution of the weight of edges becomes almost uniform in high *μ* areas, but not in low *μ* areas.

Compared with the control model, our model weakens the trade-off relationship between the edge weight distribution and mean degree. In Fig 5A, we take 200 samples from a certain instant of the EL model (Fig 5B shows the trade-off relationship for 7, 8 and 9 bits. The same tendency was observed). Both values are distributed around the mean values. The variance from the mean values is much larger than the control model. This can be observed in the time series variance of the weights in one trial (Fig 5C). In the control model, the variance of edge weights is always much smaller than the EL model. This suggests that the network structure of each parameter value remains the same through the process of one trial. Compared with the control, the variance of the weights changes dynamically in the time series of the EL model. Taking into account the correlation relationship between the mean degree and weight variance, the network structure of the EL model is not stable but dynamic.

**Fig 6:**
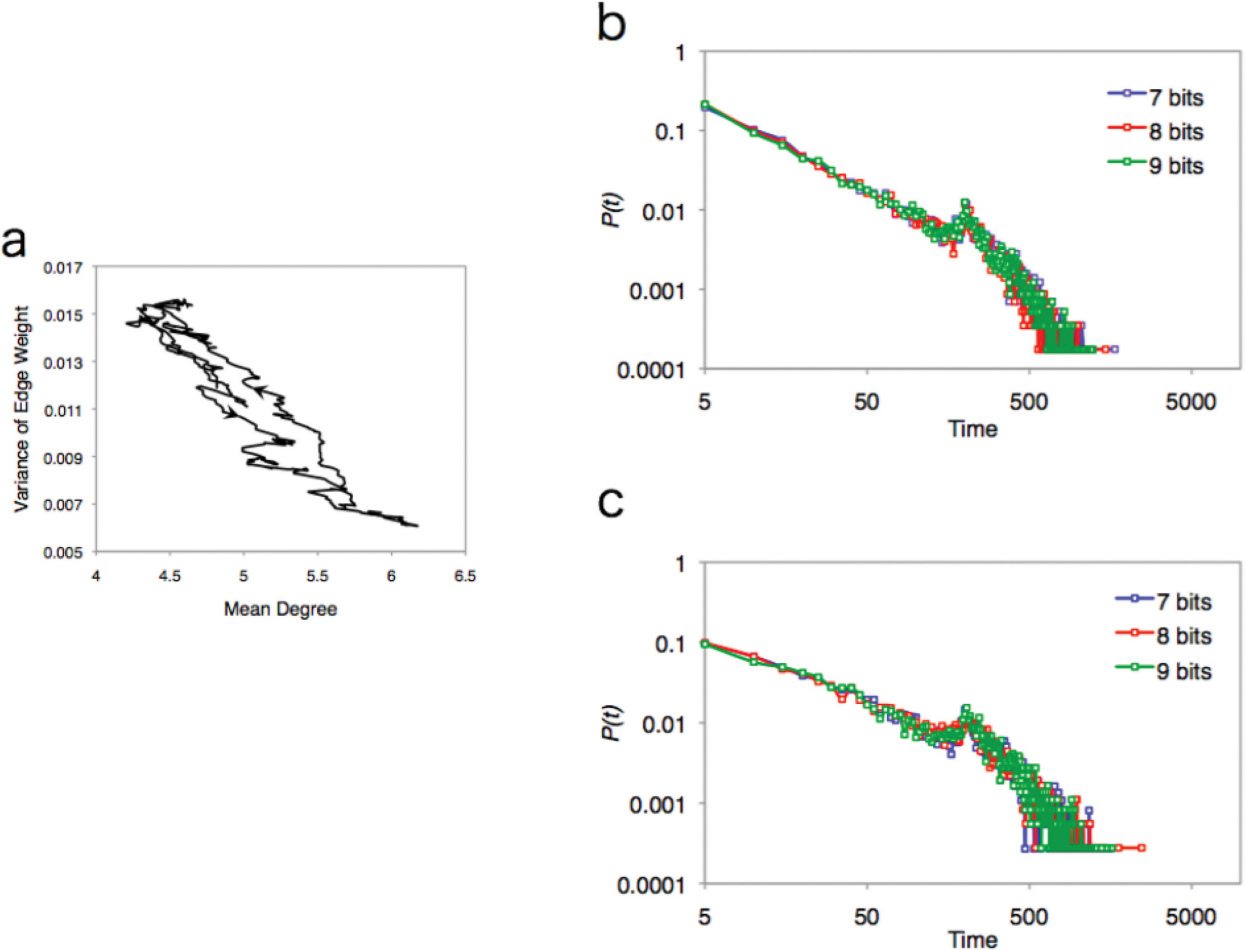
Trajectory and its recurrent time of mean degree and variance of edges weight. **(a)** Trajectory of data through 600 steps (8 bits). The arrow in the graph indicates the direction of the process. After forming a circular trajectory, the system finally returns to the initial point. **(b)** Probability distribution of the recurrent time for mean value of the mean degree. Colors correspond to number of bits (7 bits: blue, 8bits: red, 9 bit: green). The graph exhibits a power law distribution (7-bits: *N* = 119900, scaling exponent *α* = 1.39, AIC weights of power law *w*(*p*) = 1.00; 8-bits: *N* = 119800, scaling exponent *α* = 1.40, AIC weights of power law *w*(*p*) = 1.00; 9-bits: *N* = 119859, scaling exponent *α* = 1.40, AIC weights of power law *w*(*p*) = 1.00). **(c)** Probability distribution of recurrent time for mean value of the variance of edge weights. Colors correspond to the number of bits (7 bits: blue, 8bits: red, 9 bit: green). In contrast to the mean degree, the graph exhibits an exponential. (7-bits: *N* = 119900, scaling exponent *λ* = 0.031, AIC weights of power law *w*(*e*) = 1.00; 8-bits: *N* = 119800, scaling exponent *λ* = 0.031, AIC weights of power law *w*(*e*) = 1.00; 9-bits: *N* = 119859, scaling exponent *λ* = 0.031, AIC weights of power law *w*(*e*) = 1.00).

Next we examine the detail of the dynamics of the network structure of the EL model. Fig 6A shows an example of the circle trajectory of the mean degree and the weight variance (other examples are listed in S3 Fig). We take each value at each interval such as 200–400, 201–401, 202–402, and so on. This graph indicates that the mean degree in this graph gradually increases as the weight variance of the network decreases, and vice versa. In other words, the network of the EL model tries to resolve the trade-off relationship itself by tuning the tensions between the two values. This tendency reflects the recurrence time of mean value of both measurements. Fig 5B shows that it takes a long time to get back to the mean values. In particular, the probability distribution of the recurrence time for the mean degree obeys a power law distribution (see Table 3; note that *α* is not the value for the cumulative distribution). Both long time durations indicate that the EL network values have a long traveling trajectory, observed as an ellipse trajectory in Fig 6A, before returning to the same state.

**Table 3:** Data for a scaling parameter *α* of the recurrent time of power law with AIC test.

	Scaling Parameter *α*	AIC weights: *w*(*p*)
7-bits	1.39 (*N* = 119900)	1.00
8-bits	1.40 (*N* = 119800)	1.00
9-bits	1.40 (*N* = 119859)	1.00

Scaling parameter *α* and Akaike information criterion (AIC) weights of power law for *t* ^−*α*^. *t* means the recurrence time for the mean degree of an EL net- work.

## Discussion

The aim of this paper is to construct a non-ever-growing weighted network model by using fitness estimation, deduced from incomplete information. The main point of difference of our model from previous network studies is based on taking into account the internal dynamics of the network. Because of the constraint on the number of individuals (2^*n*^ individuals in total for *n* bits), the distribution of individuals is finite. Each state of an individual, which corresponds to a node, changes state in order to increase its own fitness but the number of nodes is always under 2^*n*^ because the maximum possibility of states is 2^*n*^ in a given network. This state change plays a role in the flow in this network. The network structure arises from exchanging this finite resource in a certain interval.

By taking internal dynamics in account, we are able to examine the emergence of weighted networks from local interactions. First, we found that the structure of this network (i.e. the degree distribution) in the EL model exhibits an exponential distribution instead of a power law, and we also found an asymmetrical relationship between the in-degree and out-degree distributions. These tendencies have been observed in real web networks [11]. Furthermore, we also confirmed that the weight distribution of edges obeys a power law which never depends on how the interval is taken. Although the slope of the power law is not stable, it is observed that power law behavior remains the same. All these results are good matches to previous studies [11, 16, 18]. In this respect, our assumption of internal dynamics provides sufficient evidence to say that we have a new network modeling.

Finally, we comment on the trade-off relationship between the mean degree and variance of the weight distribution discussed in the last section. We note the practical meaning of the resolution of the trade-off relationship in our model. In contrast to the control model, the network structure of the EL model dynamically changes with time. In particular, the mean degree and variance of the edge weights change in a highly correlated way. As we discussed, the high mean degree indicates the robustness of the network and the high variance of edge weights indicates the efficiency of the network. By tuning the balance between these two quantities, the network of the EL model always acts to resolve the trade-off relationship between efficiency and robustness.

We note structures that resemble ascendency theory in ecosystems. Ulanowicz measured the degree of network development by using the concept of ascendency [16, 17]. Ascendency is defined as the product of total system throughput and average mutual information. From this definition, ascendency is high when the flows in a network are distributed heterogeneously. In addition, he also noted that decreasing ascendency indicates that the number of redundant pathways (more specifically, he consider other quantities such as dissipations, inputs, and outputs) is large. This trade-off relationship between ascendency and redundant pathways corresponds to our discussion, that is, the trade-off relationship between the mean degree and variance of edge weights. Furthermore, Ulanowicz discusses the development of the network from the perspective of ascendency [17]. If the ascendency increases, then the degree of maturity of the ecosystems increases. If the ascendency of the system were to decrease, it would indicate a reset (or death) of the system and a return to an initial state.

If we accept this argument, we can insist that the EL model exhibits growth and decline of the system which resolves the trade-off relationship between robustness and efficiency because the increasing ascendency corresponds to increasing variance of edge weights and decreasing ascendency (i.e. increasing redundancy) corresponds to increasing mean degree of the network. (We examined the relation between the mean degree and variance of edge weights for real ecosystems, and found a negative correlation. Pearson’s correlation test: *N* = 15, *r* = -0.73, *p* = 0.0008. This value suggests that real networks also exhibit this trade-off relationship). Our network model is not purely dynamical, and also exhibits developmental cycles in certain time intervals. The network of the EL model gets old with time. After the network grows to a certain degree of maturity (heterogeneous structure), the network gradually decays (homogeneous structure) and prepares for the next cycle. The growing and decaying network in the EL model is one answer to the trade-off problem under the finite distribution of resources in developing systems.

## Supporting Information

S1 Fig Cumulative distribution of edge weights for a 200 step interval. Color correspond to parameter values (*μ =* 0.5: blue, *μ =* 0.05: red, *μ =* 0.5: green). Power law like behavior is only observed with high parameter values.
(EPS)

S2 Fig The relationship between R^2^ value for the least squares method for a power law. The relationship between R^2^ value for the least squares method for a power law for cumulative distribution of edge weights and the parameter *μ*. The region of high parameter values (*μ*>0.7) and of low parameter values (*μ*<0.4) are poor fits to the power law behavior.
(EPS)

S3 Fig Other examples of the trajectory of the EL model. Other examples of the trajectory of the EL model for 500 steps. All graphs show that the network of the EL model travels around the space and finally back to the initial state.
(EPS)

S1 Text Basic concept of lattice theory, the algorithm of the control model and thair analysis. (DOCX)
