# Supplementary material for: Ongoing Processes in a Fitness Network Model under Restricted Resources
Source: PLoS One. 2015 May 18;10(5):e0127284. doi: 10.1371/journal.pone.0127284 (PMC4436180; doi:10.1371/journal.pone.0127284)
Supplement: S1 Text — (DOCX) [file pone.0127284.s004.docx]

**Supporting Information of**

**“Ongoing Processes in a Fitness Network Model under Restricted Resources”**

Takayuki Niizato^*1^ and Yukio-Pegio Gunji^2^

^1^Faculty of Engineering, Information and Systems, Tsukuba University, Tsukuba, Ibaraki, Japan

^2^School of Fundamental Science and Engineering, Waseda University, Tokyo, Japan

*****Corresponding author

E-mail: [t_niizato@yahoo.co.jp](mailto:t_niizato@yahoo.co.jp)

**Basic concept of lattice theory**

Our model is constructed using lattice theory. Lattice theory is used widely in computer science such as for automata theory and system science [1, 2]. We review the basic definitions and notation which are used in our model for readers who are unfamiliar with lattice theory.

**Definition 1.1** (*Partial Order*) Let *P* be a set. An order on *P* is a binary relationship ≤ on *P* such that, for all *x*, *y*, *z* ∈ *P*

1. *x* ≤ *x*
2. *x* ≤ *y* and *y* ≤ *x ⇒ x = y*
3. *x* ≤ *y* and *y* ≤ *z ⇒ x* ≤ *z*

We denote a partially ordered set by the pair, (*P*, ≤). For example, a partial order can be constructed from a set of bit (binary) strings. A bit string *a*_1_*a*_2_*a*_3_…*a_n_* is a finite sequence of zeros and ones (*a_i_* ∈{0, 1}). An order between two bit strings such as *a*_1_*a*_2_*a*_3_…*a_n_* and *b*_1_*b*_2_*b*_3_…*b_n_* is defined by *a*_1_*a*_2_*a*_3_…*a_n_* ≤ *b*_1_*b*_2_*b*_3_…*b_n_* if *a_i_* ≤ *b_i_* for all *i*. We use a set of bit strings in this study. However, A partial order is not a lattice. We thus define *meet* and *join*. We define the join “∨” and meet “∧” of two elements *x* and *y* in *P.* The join is defined by *x*∨*y =sup*{*x*, *y*} when it exists. The meet is defined by *x*∧*y =inf*{*x*, *y*} when it exists. The notation *sup* (*inf*) means the lowest (greatest) upper bound of {*x*, *y*} in *P*.

In particular, when elements are bit strings, meet and join can be more precisely described as follows.

**Definition 1.1.2** (*Meet and Join of n-Bit String*) Let 〈*a*〉*_n_* and 〈*b*〉*_n_* be *n*-bit strings.

(*meet*) 〈*a*〉*_n_* ∧ 〈*b*〉*_n_*:= min{*a*_1_, *b*_1_} min{*a*_2_, *b*_2_}…min{*a_n_*, *b_n_*}

(*join*) 〈*a*〉*_n_* ∨ 〈*b*〉*_n_*:= max{*a*_1_, *b*_1_} max{*a*_2_, *b*_2_}…max{*a_n_*, *b_n_*}

The notation max*S* (min*S*) indicates the largest (smallest) element in a set *S*.

**Definition 1.2** (*Lattice*) Let (*P*, ≤) be a non-empty partially ordered set.

If *x*∨*y* and *x*∧*y* exist for all *x*, *y* ∈ *P*, then (*P*, ≤) is called a lattice.

To distinguish them from a partially ordered set, we denote lattices by (*L*, ≤, ∧, ∨). In this paper, there are often-used sets: an ideal and a filter. An ideal is used when we construct the congruence on a lattice.

**Definition 1.3** (*Ideal*) Let (*L*, ≤, ∧, ∨) be a lattice. A non-empty subset of *J* is called an *ideal* if

(i) *x*, *y* ∈ *J* implies *x*∨*y* ∈ *J,*

(ii) *x* ∈ *L ,* *y ∈ J* and *x* ≤ *y* imply *x* ∈ *J.*

**Definition 1.4** (*Filter*) Let (*L*, ≤, ∧, ∨) be a lattice. A non-empty subset of *F* is called a *filter* if

(i) *x*, *y* ∈ *F* implies *x*∧*y* ∈ *F,*

(ii) *x* ∈ *L ,* *y* ∈ *F* and *y* ≤ *x* imply *x* ∈ *F.*

The typical example of an ideal is a down set on a lattice. The definition of a down set is a subset *J* = {*y* *∈ L* | *y* ≤ *x*} when *x* ∈ *L*. We denote a down set of *x* as ↓*x*. It can easily be verified that a down set satisfies the conditions of an ideal. In a similar way, we can define an up set on a lattice such as *F* = {*y ∈ L* | *x* ≤ *y*} when *x* ∈ *L*. We denote an up set of *x* as ↑*x*. We can also verify that an up set satisfies the conditions of a filter. Next, we consider congruence on a lattice to define a quotient lattice. A congruence is an equivalence relation which is restricted by a certain condition.

**Definition 1.5** (*Congruence on a Lattice*) Let (*L*, ≤, ∧, ∨) be a lattice. Let an equivalence relation on *L* be *θ* = {<*x*,*y*> ∈ *L*×*L*} such that any *x*, *y*, *z* ∈ *L*,

1. <*x*,*x*> ∈ *θ*
2. <*x*,*y*> ∈ *θ ⇔* <*y*,*x*> ∈ *θ*
3. <*x*,*y*> ∈ *θ* and <*y*,*z*> ∈ *θ* ⇒ <*x*,*z*> ∈ *θ*

We also denote <*x*,*y*> ∈ *θ* by *x*≡*y* (mod *θ*). An equivalence relation is thus a congruence on *L*, if for any *x*, *y*, *z, w* ∈ *L*, ( *x*≡*y* (mod *θ*) and *z*≡*w* (mod *θ*) ) ⇒ ( *x*∨*z* ≡ *y*∨*w* (mod *θ*) and *x*∧*z* ≡ *y*∧*w* (mod *θ*) )

We can then make a quotient lattice by using a congruence.

**Definition 1.6** (*Quotient Lattice*) Let *θ* be a congruence on a lattice (*L*, ≤, ∧, ∨), then a set *L*/*θ* is defined by

*L*/*θ =*{[*x*]*_θ_* | *x* ∈ *L*} with [*x*]*_θ_* = {*y* ∈ *L* | *x*≡*y* (mod *θ*)}

The join and the meet on *L*/*θ* are defined by

[*x*]*_θ_* ∧ [*y*]*_θ_* := [ *x*∧*y* ]*_θ_* , [*x*]*_θ_* ∨ [*y*]*_θ_* := [ *x*∨*y* ]*_θ_*

We call (*L*/*θ*, ≤, ∧, ∨) the quotient lattice of *L* modulo *θ*.

In this study, we construct a quotient lattice from a given ideal. First we introduce the equivalence relation derived from an ideal.

**Definition 1.7** (*Equivalence Relation Derived from an Ideal*) Let *J* be an ideal on a lattice *L.* The equivalence relation derived from an ideal *J* is

*θ* (*J*):={<*x*, *y*> ∈ *L*×*L*| ∃*z* ∈ *J* *x*∨*z* = *y*∨*z* }

We can easily verify that Definition 2.7 satisfies Definition 2.5 and that *θ* (*J*) is well-defined to be a congruence relation. The next proposition ensures that the ideal is a block of the partition derived by a quotient lattice. This proposition will be used when we discuss the existence of the lowest block information.

**Proposition 1.8** Let *θ* (*J*) be an equivalence relation derived from an ideal *J* on a lattice *L*. Then *J* is a block of the corresponding partition of *L*.

**Proof.** *J* is a block of the corresponding partition of *L* iff ∀ *x* ∈ *J*, [*x*]*_θ_* (*_J_*) = *J* (*)

Therefore we must prove (*).

1. ∀ *y* ∈ [*x*]*_θ_* (*_J_*) ⇔ (∃*z ∈ θ (J)*) *x*∨*z* = *y*∨*z* ⇒ *x*∨*z* = *y*∨*z* ∈ *J* (Definition 2.3) ⇒ *y*≤*y*∨*z* , *y* ∈ *J* (Definition 2.3)
2. Supposing *y* ∈ *J,* for [*x*]*_θ_* (*_J_*), it is trivial that *x* ∈ *J*, then *x*∨*y* ∈ *J*. Clearly, *x*∨(*x*∨*y*) = *y*∨(*x*∨*y*). It entails *y* ∈ [*x*]*_θ_* (*_J_*)

From (i) and (ii), [*x*]*_θ_* (*_J_*)=*J* is proved.

We also point out that a quotient lattice is deeply connected to a map between lattices, which is called a *homomorphism*.

**Definition 1.9** (*Homomorphism*) Let *L* and *K* be a lattices. A map *f* : *L* → *K* is said to be a homomorphism if for any *x*, *y* ∈ *L*,

1. *f* (*x*∧*y*) = *f* (*x*) ∧ *f* (*y*) (*meet-preserving*)
2. *f* (*x*∨*y*) = *f* (*x*) ∨ *f* (*y*) (*join-preserving*)

Especially, *f* is called isomorphism when *f* is a bijective homomorphism.

**Proposition 1.10** (*Homomorphism between a Lattice and a Quotient Lattice*) Let *θ* be a congruence on a lattice *L*. Then (*L*, ≤, ∧, ∨) is a lattice and a natural quotient map *f* : *L* → *L*/*θ*, defined by *f* (*x*) := [*x*]*_θ_* , is a homomorphism.

**Proof.** We check that a natural quotient map *f* satisfies Definition 2.5.

*f* (*x*∨*y*) *=*[ *x*∨*y* ]*_θ_* = [*x*]*_θ_* ∨ [*y*]*_θ_* = *f* (*x)* ∨ *f* (*y*)

*f* (*x*∧*y*) *=*[ *x*∧*y* ]*_θ_* = [*x*]*_θ_* ∧ [*y*]*_θ_* = *f* (*x)* ∧ *f* (*y*)

The next theorem is often used in this paper. The theorem is used for constructing a quotient lattice from an ideal. Using this theorem, we can determine a quotient lattice from a given lattice.

**Theorem 1.11** (*Reconstruction of a Lattice from a Quotient Lattice*) Let *L* be a lattice and *f* be a natural quotient map such as *f* : *L* → *L*/*θ.* For the binary relation derived from an ideal *J* ⊆ *L*, there exists a filter *K* ⊆ *L* such that [*x*]*_θ_* (*_J_*) = *f* ^-1^(*x*), where for any *x* ∈ *K*, *f* ^-1^(*x*) := ↓*x* - ∪*_y_* _∈_ *_K, y<x_*↓*y*

**Proof.** See [2].

**Detail of EL algorithm**

STEP 1. Generate 2*^n^* *n*-bit strings 〈*s*〉*_n_*^1,0^,〈*s*〉*_n_*^2,0^, …〈*s*〉*_n_^N^,*^0^ and a target *n*-bit string 〈*t*〉*_n_* randomly. The target is fixed through one trial. A fixed target is an environment. An environment changes much slower than the transitions of an individual.

STEP 2. Randomly select a hidden digit of the target from the set {*t*_1_, *t*_2_, …, *t_n_*}, say *t_i_*. Each individual can observe *t*_1_*t*_2_…*t_i_*_-1_*t_i+_*_1_…*t_n_*. A fitness is formed from a set of *n*-bit strings *B ^t^* = {〈*b*〉*_n_*^1^*^,t^*, 〈*b*〉*_n_*^2^*^,t^*, …, 〈*b*〉*_n_^N,t^*} using Definition 1.1. *N* = 2*^n^*.

STEP 3. Create a lattice *L^t^* from this *B^t^*. Note |*L^t^* |(= *M*) >*N* because we need to add top and bottom elements at the least. We denote this lattice {〈*c*〉*_n_*^1^*^,t^*, 〈*c*〉*_n_*^2^*^,t^*, …, 〈*c*〉*_n_^M,t^*}. Select one element 〈*c′*〉*_n_^t^* in the lattice *L^t^* and create the ideal ↓〈*c′*〉*_n_^t^*.

STEP 4. Construct a quotient lattice, denoted $L^{t}/\theta(J)=\{[\left\langle c\right\rangle_{n}^{1,t}]_{\theta(J)},[\left\langle c\right\rangle_{n}^{2,t}]_{\theta(J)},...,[\left\langle c\right\rangle_{n}^{M',t}]_{\theta(J)}\}$, from the ideal *J* =↓〈*c′*〉*_n_^t^*. Note $|L^{t}/\theta(J)|=M'(\leq M)$.

STEP 5. Substitute new binary bit strings of fitness.

$\left\langle b\right\rangle_{n}^{i,t}\leftarrow\vee\{\left\langle x\right\rangle_{n}^{k,t}\in L^{t}\left|\left\langle x\right\rangle_{n}^{k,t}\in[\left\langle c\right\rangle_{n}^{j,t}]_{\theta(J)}\text{ such that }\right.\left\langle b\right\rangle_{n}^{i,t}\in[\left\langle c\right\rangle_{n}^{j,t}]_{\theta(J)}\}$.

STEP 6. Change each *n*-bit string (state) using fitness. For 1≤*j*≤*n*,

$$s_{j}^{i,t+1}=\left\{\begin{matrix} s_{j}^{i,t}\text{ (if }b_{j}^{i,t}\text{=1)} \\ \text{0 or 1 (otherwise)} \end{matrix}\right.$$

Construct a new set of binary bit strings for species *B^t^*^+1^ = {〈*s*〉*_n_*^1^, *^t^*^+1^, 〈*s*〉*_n_*^2^, *^t^*^+1^, …, 〈*s*〉*_n_^N^*, *^t^*^+1^}.

STEP 7. Go back to STEP 2.

**Detail of Control Model**

STEP 1. Generate 2*^n^* *n*-bit strings 〈*s*〉*_n_*^1,0^,〈*s*〉*_n_*^2,0^, …〈*s*〉*_n_^N^,*^0^ and a target *n*-bit string 〈*t*〉*_n_* randomly. The target is fixed through one trial. A fixed target is an environment. An environment changes much slower than transitions of an individual.

STEP 2. Randomly select a hidden digit of the target from the set {*t*_1_, *t*_2_, …, *t_n_*}, say *t_i_*. Each individual can observe *t*_1_*t*_2_…*t_i_*_-1_*t_i+_*_1_…*t_n_*. A fitness is formed from a set of *n*-bit strings *B ^t^* = {〈*b*〉*_n_*^1^*^,t^*, 〈*b*〉*_n_*^2^*^,t^*, …, 〈*b*〉*_n_^N,t^*} as follows. *N* = 2*^n^*.

$$b_{j}^{i,t+1}=\left\{\begin{matrix} \text{1 (}s_{j}^{i,t}\text{=}s_{j}^{i,t}\text{)} \\ \text{0 or 1 (otherwise)} \end{matrix}\right.$$

STEP 3. Change each *n*-bit string (state) using fitness. For 1≤*j*≤*n*,

$$s_{j}^{i,t+1}=\left\{\begin{matrix} \left\{\begin{matrix} s_{j}^{i,t}\text{ (if }b_{j}^{i,t}\text{=1 with the probability }\mu\text{)} \\ 0\text{ or }1\text{ (if }b_{j}^{i,t}\text{=1 with the probability 1-}\mu\text{)} \end{matrix}\right. \\ \text{0 or 1 (otherwise)} \end{matrix}\right.$$

Construct a new set of binary bit strings for species *B^t^*^+1^ = {〈*s*〉*_n_*^1^, *^t^*^+1^, 〈*s*〉*_n_*^2^, *^t^*^+1^, …, 〈*s*〉*_n_^N^*, *^t^*^+1^}.

STEP 4. Go back to STEP2.

**Behavior of the control model for parameter tuning**

We take the mutation parameter *μ* (error rate) from 0.005 to 1 (binned 0.005). Tuning the parameter value induces qualitatively different behavior in the network. In the main text, we discuss a directed network. How about the edge weight distribution? S1 Fig. shows three distributions of the edge weights. The graph suggests that power law behavior never emerges for low error rates *μ* such as 0.005 and 0.05. Although the network exhibits power law behavior in the edge distribution like the EL model, this graph corresponds to an exponential rather than a power law distribution (*N*=119900, scaling exponent *λ*= 0.031, AIC weights of exponential *w*(*e*)=1.00). As we saw in Fig. 5a, the mean degree of the network, that is, *μ* =0.5, is much larger than that of the EL model, whereas the variance of edge weights is much smaller than the EL model. In this sense, the control model not only fails to resolve the trade-off relationship, but also fails to distribute the resources to each node. We added the graph the power-law like behavior is only located on high parameter value (0.4 ≤*μ*≤0.7: see S2 Fig.).

**Reference**

1. Davey BA and Priestelely HA (2005) Introduction to Lattices and Oder. Cambridge. Cambridge University Press.
2. Gunji YP, Haruna T and Sawa K (2006) Principles of Biological Organization: Local-Global Negotiation Based on “Material Cause”. Physica D. 219. pp. 152-167.
3. Clauset A, Shalizi CR and Newman ME (2009) Power –Law Distribution in Empirical Data. SIAM Review. 51. 4. pp. 661-703.

Edwards AM, Phillips RA Watkins NW, Freeman MP, Murphy EJ, et al. (2007) Revisiting Lévy Flight Search Patterns of Wandering Albatrosses, Bumblebees and Deer. *Nature* **449**, 1044 – 1049.
